# *Centella asiatica* (Gotu kola) ethanol extract up-regulates hippocampal brain-derived neurotrophic factor (BDNF), tyrosine kinase B (TrkB) and extracellular signal-regulated protein kinase 1/2 (ERK1/2) signaling in chronic electrical stress model in rats

**DOI:** 10.22038/ijbms.2019.29012.7002

**Published:** 2019-10

**Authors:** Dwi Cahyani Ratna Sari, Nur Arfian, Untung Tranggono, Wiwit Ananda Wahyu Setyaningsih, Muhammad Mansyur Romi, Noriaki Emoto

**Affiliations:** 1 Department of Anatomy, Faculty of Medicine, Public Health and Nursing, Universitas Gadjah Mada, Yogyakarta, Indonesia; 2 Department of Surgery, Faculty of Medicine, Public Health and Nursing, Universitas Gadjah Mada, Yogyakarta, Indonesia; 3 Division of Cardiovascular Medicine, Department of Internal Medicine, Kobe University Graduate School of Medicine, Kobe, Japan; 4 Laboratory of Clinical Pharmaceutical Science, Kobe Pharmaceutical University, Kobe, Japan

**Keywords:** BDNF signaling, Chronic electrical stress – model, Centella asiatica, ERK1/2, TrkB

## Abstract

**Objective(s):**

Impairment of hippocampus function as a center for memory processing occurs due to stress. *Centella asiatica* L. (Gotu kola) is known to improve memory, intelligence, and neural protection although the precise mechanism is not well understood. This study aimed to investigate the effects of ethanol extracts of *C. asiatica* toward MAPK expression as down-stream signaling of brain-derived neurotrophic factor (BDNF).

**Materials and Methods:**

We performed a chronic electrical stress model on 20 male Sprague Dawley rats (2 months-old, 180–200 g). Rats were divided into four groups: normal control group (Control) which received distilled water, and three treatment groups receiving oral Gotu kola ethanol extracts in oral doses of 150 mg/kg BW (CeA150), 300 mg/kg BW (CeA300), and 600 mg/kg BW (CeA600) over four weeks. Memory acquisition was assessed with Morris water maze. Hippocampus was harvested, then extracted for protein and RNA analysis. MAPK proteins (p38, ERK1/2, JNK) were measured using Western blot, meanwhile, BDNF and TrkB receptor were analyzed with real-time PCR (RT-PCR).

**Results:**

CeA600 group revealed improvement of memory performance as shown by reduction in time and distance parameters compared to control during escape latency test. This finding associated with significant elevation of hippocampal BDNF protein and mRNA level with up-regulation of TrkB mRNA expression in CeA600 group compared to control. Western-blot analysis showed significant up-regulation of ERK1/2 protein level in CeA600 group (*P*<0.05) compare to control.

**Conclusion:**

BDNF signaling through TrkB and ERK1/2 pathway contributes significantly to amelioration of memory performance after *C. asiatica* treatment in electrical stress model.

## Introduction

Life may contain stresses to human, ranges from genetics to environments. Chronic stress may cause negative effects for mental and physical health (1). The body will actively respond to stress and disturb biological function then gives an impact to the brain (2). Stress may impact to hippocampus, which provides the gateway of our learning, then it spreads to other interconnected brain regions, such as amygdala and prefrontal cortex. This condition may be influenced by glucocorticoid or adrenal steroid (3). Many studies showed that stress disturbs hippocampal-dependent memory tasks, changes hippocampal synaptic plasticity and firing properties. Stress also affects neuronal morphology, neuron’s proliferation inhibition and hippocampal volume reduction (4). 

Memory formation begins with the formation of Long-Term Potentiation (LTP) and changes in brain morphology such as neuropil growth and synaptogenesis (5). Recently, it has been postulated that brain-derived neurotrophic factor (BDNF) has an important role in promoting neuronal survival, differentiation, synaptic plasticity which important for learning and memory mechanisms in the adult CNS (6,7). In addition, there is a correlation between BDNF mRNA expression with the behavior in a variety of learning and memory tests such as the Morris water maze test (5). Continuous infusion of intra-cerebrovascular antisense BDNF oligonucleotides has proven to inhibit the synthesis of BDNF in rats along with decreased function in the maze test. This finding shows the importance of BDNF synthesis in the process of spatial memory formation, as well as memory retention and recall (8).

The influence of stress on BDNF levels leads to different results. According to Xiao-heng (2007), there was an increase in BDNF mRNA expression in the hippocampus of rats with increased learning and memory capacities after multiple chronic stress (9). Other studies have shown that exposure to chronic mild stress in rats caused a decrease in BDNF mRNA expression in the dentate gyrus but not in other parts of the hippocampus (10). Exposure to acute stress in rats led to significant increase of BDNF in the hippocampus to both the young and old age groups, whereas chronic mild stress caused a decrease in BDNF protein in both groups (11).

The use of *Centella asiatica *L. (Gotu kola) to improve memory, intelligence, and neural protection has long been known. The active pentacyclic-triterpenoid-saponins in *C. asiatica* such as asiaticoside and madecoside are believed to improve memory. According to Ramaswamy (2005), memory improvement is expectedly due to the decreased alteration of central monoamines involving norepinephrine and the 5-hydrotetraamine system (5-HT) (12). Asiaticoside prevents cell death and apoptotic on N-methyl-D-aspartate (NMDA)-induced excitotoxicity in cultured cortical neuron (13). Additionally, the asiatic acid is known to accelerate the repair of damaged neurons through axon regeneration and neurite growth (14). Recently, it was reported that *C. asiatica* supplementation itself could improve memory performance by inducing serum BDNF level and reducing nitrite oxide (NO) level in rats with chronic electrical stress (15). Activation of the BDNF-receptor complex induced down-stream signaling pathways mediated by mitogen-activated protein kinase (MAPK) as a transcription factor. The relationship between learning and memory with MAPK has been widely reported (16, 17), but its effect on memory retention changes have not been widely discussed. Therefore, we aimed to elucidate the effects of ethanol extracts of, *C. asiatica* toward MAPK (p38 protein, ERK1/2, and JNK) expression as down-stream signaling of BDNF and its association with memory retention in the rat hippocampus after treatment of electrical stress. 

## Materials and Methods

***Animals and treatments***

The subjects of this study were 20 male Sprague Dawley rats which weighed about 180-200 g and were obtained from the Experimental Animal Care Unit (UPHP) of Universitas Gadjah Mada. The 20 rats were housed in groups inside glass cages with each cage containing 1 rat. Before grouping, the rats were given identities by cutting their ears with modifications of rat identification with a randomized method. Every day the rats were given food pellets and water *ad libitum*. The conditions were expected to be room temperature of 25-30 ˚C with 50%-60% humidity and a dark-light cycle of 12:12 hrs. The rats were given 7 days to adapt to the environment of the cages. All experimental procedures described were approved by the Medical and Health Research Ethics Committee (MRHEC), Faculty of Medicine, Public Health and Nursing, Universitas Gadjah Mada, Yogyakarta, Indonesia with number Ref. KE/FK/657/EC.

The subjects were randomly divided into 4 groups of 5 rats in each group. The groups were the normal control group which only received distilled water as the solvent of the ethanol extract, and treatment groups which received ethanol extracts of *C. asiatica *(CeA) leaves orally. The groups were given CeA leave ethanol extract doses of 150 mg/kg body weight (CeA150 group), 300 mg/kg body weight (CeA300 group), and 600 mg/kg body weight (CeA600 group) with concentrations of 30, 60, and 120 mg/ml. 

Chronic electrical stress model was performed to induce memory disturbance in rats based on the previous study (11). Briefly, electrical stress was given by inserting a rat into a plexiglass box with an electrical current of 0.8 mA and shocking the rat for 5 sec every 15 sec (three times per minute, 5 sec per shock), for a total treatment 10 mins. The rats were fully supervised throughout the treatment to prevent any undesired effects and were weighed on days 0, 14, and 28.

***Herbal solution preparation***

*C. asiatica *leaves were obtained from the commercial herb manufacturer Merapi Farma Herbal, Kaliurang, Sleman, Yogyakarta. The *C. asiatica *were then identified and authenticated at the Faculty of Mathematics and Natural Sciences, Universitas Gadjah Mada. The *C. asiatica* leaves were macerated with 70% ethanol (the absolute ethanol was solved in distilled water) at the Integrated Research and Testing Laboratory (LPPT) of Universitas Gadjah Mada. 

***Hidden platform test (escape latency)***

Escape latency test was performed to examine memory acquisition function as part of Morris water maze (MWM) experiments. The MWM test in this study was continuation of MWM test from our previous study (11). It was done twice during the experiment, before inducing stress for 6 days (pre-treatment) and after inducing stress and CeA treatment for 6 days (post-treatment). Rat’s memory was assessed based on 2 parameters, time and distance. In this experiment, hidden platform was placed in the targeted quadrant of the MWM. We randomly placed the rats in the pool and observed the rats during finding platform. Time allocation of the rats to find the platform was quantified. Using video recording we quantified distance of the rats from starting point to the platform. Data were expressed in second as unit for time parameter and meter as unit for distance parameter. 

***Isolation of hippocampus and protein extraction for ELISA***

One hour after the last execution of the acquisition test, rats were sacrificed, then hippocampus tissue was isolated manually and stored at a temperature of -80 ˚C. Protein extraction was performed by the adding 600 µl of PRO-Prep^TM^ protein extraction solution (containing protease inhibitors: PMSF, EDTA, Pepstatin A, Leupeptin, and Aprotinin) to 10 mg of hippocampus tissue. The hippocampus was then homogenized with a tissue protein extraction solution using a cup and mortar in a cold atmosphere. Once it was homogeneous, the tissue lysates were incubated at -20 ˚C for 30 min followed by centrifugation of 12.000 rpm for 10 min at 4 ˚C. The supernatant from the hippocampus tissue lysates was removed from the pellets and then stored in 1.5 ml tubes at -80 ˚C. Protein was used for BDNF ELISA based on protocol from the Rat BDNF ELISA kit (Boster Immunoleader, Cat. EK0308). 

***Western-blotting***

The ELISA was followed by Western blot using the same protein. A total of 40 μg of protein was separated into 8% SDS-PAGE, and transferred to a polyvinylidene fluoride membrane (PVDF) and incubated with anti-p38 (sc535, Santa Cruz), anti-ERK1/2 (sc93, Santa Cruz), and anti-JNK1 (sc571, Santa Cruz) antibodies. The GAPDH (7074, Cell Signaling) was used for the expression normalization. A total of 5% skim milk in TBST was used for blocking followed by incubation with the appropriate secondary antibody. Proteins were visualized using a Luminata Forte Western HRP Substrate (Millipore, WBLUF0100). Blots were photographed with a transilluminator LAS 3000 mini (Fuji Film) and quantified with densitometry using Image Reader LAS 3000 mini.

***RNA extraction, cDNA making and real time PCR (RT-PCR)***

Left hippocampus was used for RNA extraction and RT-PCR. Briefly, the hippocampus was kept in RNA Later after harvesting, homogenized in Trizol (Invitrogen, 1559-018, Paisley, UK) for RNA extraction. The cDNA was made based on manufacturer instruction using Rever-TraAce (Toyobo, TRT 1001). The cDNA was used for RT-PCR quantification for BDNF and TrkB expression in the hippocampus. These following primers were used: BDNF forward (ATGACCATCCTTTTCCTTACTATGGT), BDNF reverse (TCTTCCCCTTTTAATGGTCAGTGTAC); TrkB forward (AGCCTTCTCCAGGCATCGT), TrkB reverse (CGGGTCAACGCTGTTAGGTT); and GAPDH forward (GGCACAGTCAAGGCTGAGAATG), GAPDH reverse (TCTCGCTCCTGGAAGATGGTGA) for housekeeping gene. The RT-PCR was performed using Thunderbird SyBR reagent (Toyobo, QPK-101).

***Data analysis***

To determine the normality of the data distribution, the Kolmogorov-Smirnov test or Shapiro-Wilk was used. The results of these tests determined whether the research data should be tested with parametric or nonparametric statistical tests. The data were analysed using one-way ANOVA to determine statistically significant differences between BDNF levels in each group, followed by *Post hoc-*Least Significant Difference (LSD). For memory performance, we used General Linear Model-Repeated Measurement (GLM-RM).

## Results

***Escape latency test of MWM before and after treatments***

There were no different in time and distance parameters between all groups in pre-treatment results of escape latency test. It showed that the basic memory functions of the rats were homogenous. Meanwhile, post-treatment results showed significant different in day 5 and day 6 in time parameter and day 1, day 2, day 5 and day 6 in distance parameter. Reduction of time and distance during escape latency test showed role of learning in this process. However, we found significant lower in time parameter between control and CeA groups in day 5 and day 6. It demonstrated effect of CeA in amelioration of acquisition memory. These results were confirmed with distance parameter of post-treatment in day 5 and day 6. These results showed reduction of time and distance that were needed for reaching the targeted platform in CeA groups. CeA600 group had the lowest distance in post-treatment of escape latency test which demonstrated this group had the highest memory performance among others.

***BDNF concentration and expression associated with TrkB expression in the hippocampus***

**Figure 1:**
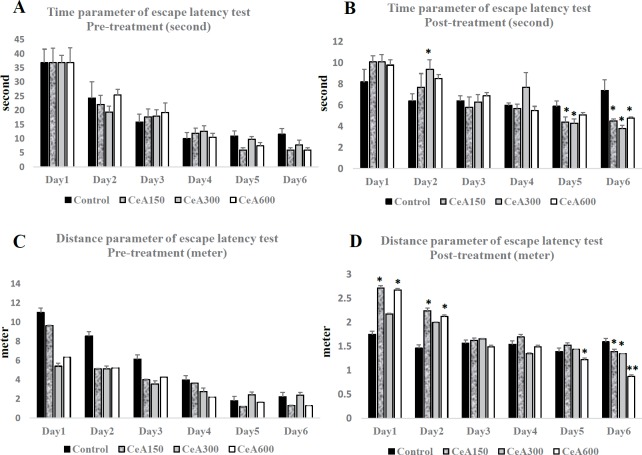
Comparison of escape latency test in Morris water maze before (pre-treatment) and after (post-treatment) chronic electrical. A-B. Pre-treatment and post-treatment of time parameter of escape latency test. C-D. Pre-treatment and post-treatment of distance parameter of escape latency test. Data were expressed as Mean±SEM. * *P*<0.05 vs Control group in same day trial; **P<0.01 vs Control group in same day trial

The average BDNF (pg) concentration per milligram of hippocampus tissue after the chronic electrical stress of the Control group (359.28±36.18), CeA150 (432.34±24.65), CeA300 (437.13±21.76), and CeA600 (497.01±27.44) (*P*<0.05) are shown in Figure 1. The RT-PCR result also confirmed upregulation of BDNF expression after CeA treatment. The CeA300 and CeA600 groups showed significant difference compared to the control group. The up-regulation of BDNF concentration and expression associated with TrkB expression also showed that TrkB might facilitate BDNF signalling. The CeA300 and CeA600 had significant higher TrkB expression compared to the control. 

***MAPK (p38 protein, ERK1/2, and JNK) expression in the hippocampus***

**Figure 2:**
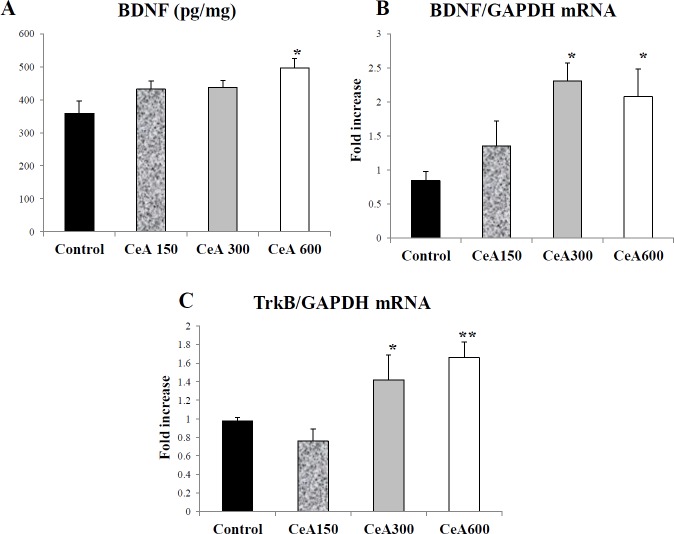
The concentration of BDNF (pg/mg), the expression of BDNF mRNA, and the expression of TrkB mRNA in hippocampal tissue after* Centella asiatica* (CeA) treatment of chronic electrical stress in all four groups (*P*<0.05, each group n=5). (A) The hippocampal BDNF protein level was assessed by ELISA (B-C) The mRNA expression of hippocampal TrkB and BDNF (each group n=5). Data were expressed as Mean±SD. *= *P*<0.05 vs Control, **= *P*<0.05 vs CeA150 group. CeA150, CeA300 and CeA600 represented groups with CeA 150, 300 and 600 mg/Kg Body weight treatment

Next, we investigated the contribution of MAPK pathways in the upregulation of BDNF-TrkB expression in the hippocampus. We analyzed protein expression of GAPDH (37 kDa) as housekeeping protein and MAPK protein which consists of ERK1/2 (42 kDa and 44 kDa), p38 (43 kDa), and JNK (46/56 kDa) using immunoblotting. The result demonstrated significantly higher expression of the ERK1/2 protein in the CeA600 group compared to the control (*P*<0.05, Figure 2), whereas the p38 and JNK pathways did not display any significant differences. Increased MAPK/ERK kinase activity was mainly found in the CeA600 group.

CeA150, CeA300 and CeA600 represented groups with CeA 150, 300 and 600 mg/Kg Body weight treatment

**Figure 3:**
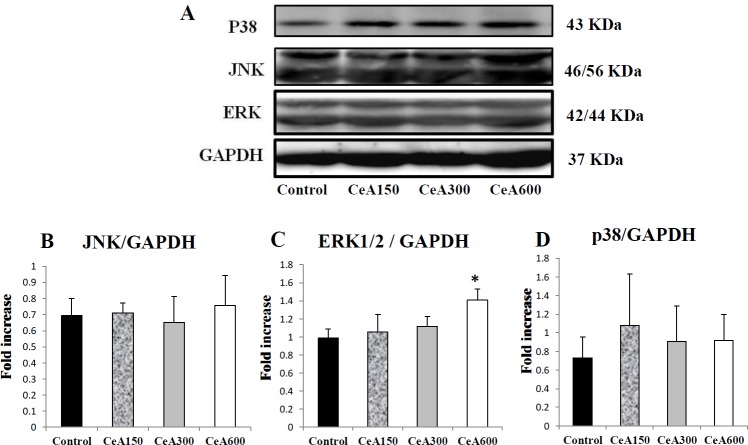
The expression of MAPK in the hippocampus tissues after administration of ethanol extract of *Centella asiatica (CeA) *after chronic electrical stress. (A) The representative picture of p38, JNK, ERK, and GAPDH analyzed with western blotting (B-D) The semi-quantitative protein result of the Western blotting analysis. Data were expressed as Mean±SD. * *P*<0.05 vs Control group (each group n=4-5). CeA150, CeA300 and CeA600 represented groups with CeA 150, 300 and 600 mg/Kg Body weight treatment

## Discussion

Here we report that ethanol extracts of *C. asiatica* improved BDNF expression and its down-stream signalling through TrkB-MAPK pathways in the hippocampus. The up-regulation of BDNF/TrkB signalling associated with improvement of acquisition memory based on escape latency test of MWM experiment. These result also supported our previous study that showed amelioration of memory performance after CeA treatment was associated with serum BDNF level up-regulation after chronic stress (13, 16). Escape latency test showed that our pre-treatment data were homogenous. Meanwhile, post-treatment data showed amelioration of memory due to learning of the rats. However, this test also revealed CeA groups had lower time and distance parameters in escape latency test. CeA600 group had the highest improvement in the test with lowest time and distance parameters in day 6 of post-treatment of escape latency test. 

Administration of ethanol extract of CeA with varying doses increased the BDNF concentration in the hippocampus tissue (Figure 2A-B). Hippocampus contains glucocorticoid receptor and glutamate and regulates hypothalamus-pituitary-adrenal (HPA) axis which susceptible to stress. Stress impairs hippocampus-dependent explicit memory and changes in synaptic plasticity in animal model (19). Our previous report revealed CeA increased memory function and is associated with BDNF level in serum (15). It seems CeA could increase memory function and serum BDNF level through up-regulation of BDNF protein and mRNA expression in the hippocampus, as shown in our result (Figure 2). Memory formation involves short-term changes in the brain’s electrical properties as well as long-term changes in the structure of the synapse. Long-term potentiation (LTP) in the hippocampus is an activity-dependent modification on the synapse strength which is the basis of learning and memory (16). The BDNF plays an active role in modulating the strength of synapses in the learning and memory processes. The BDNF promotes synaptic growth and formation in dose-dependent manner (20) as shown by BDNF involvement in the learning and memory and decreasing learning ability due to interruption of BDNF expression using the genetic approach or BDNF (21). The neuroprotective effect of BDNF involves the suppression of oxidative stress. Mice with deficiency of the BDNF showed the increment of lipid peroxidation as well as malondialdehyde (MDA) and catalase enzyme under the acute restraint stress condition (22).

The *C. asiatica* inhibits the production of the inducible nitric oxide synthase (iNOS), cyclooxygenase-2 (COX-2), as well chemokine and cytokine proinflammatory through nuclear factor kappa-B (NFκB) (23–25). Stress is associated with immune system dysfunction that influences inflammatory mediators. It is triggered by activation of reactive oxygen species (ROS) through activation of NFκB (26). Chronic stress induces oxidative stress through cyclooxygenase-2 (COX-2) activation (27). The *C. asiatica* has neuroprotective compound due to inhibition of COX-2 expression. Improvement of BDNF level in hippocampus might indicates a protective effect of CeA. The administration of *C. asiatica* ameliorates oxidative stress both *in vitro* and *in vivo* experimental models. Furthermore, the Centella asiatica which contains flavonoids and polyphenols inhibit reactive oxygen species (ROS) (28). *C. asiatica* induces a decrease in the brain level of MDA with simultaneous significant increase in glutathione and catalase levels. Thus it involves antioxidant mechanisms in preventing cognitive deficits (29, 30). Asiatic acid and asiaticoside has been shown as anti-apoptotic effect and stabilized mitochondrial membrane potential (MMP) in neuron cells (13, 31). Madecoside one of the CeA compounds have beneficial effects in rats after 1-methyl-4-phenyl-1,2,3,6-tetrahydropyridine (MTTP) induction as the initial phase of Parkinson’s disease. Madecoside increased dopamine, the ratio of Bcl-2/Bax and expression of BDNF protein. This result demonstrated that *C. asiatica* was also effective as an antioxidant and anti-apoptosis agent (32). The effect of CeA in memory function might be mediated by its active compounds or the CeA extract itself. It is beyond the aims of this study to differentiate it. It has been reported that the capability of CeA to increase memory function was mediated by its active compounds, especially asiatic acid, to cross the blood-brain barrier (33). Active penta-cyclic-triterpenoid-saponin compounds in *C. asiatica *such as asiatic acid and asiaticoside have effects on improving cognition and are efficacious as antioxidants (34). Asiaticoside accelerates axonal regeneration and plays a role in repairing damaged nerve cells, both in the size and number of myelinated axons (14). Gotu kola extracts are proven to increase dendrite arborization in the CA3 subregions of the hippocampus during the development of neonatal rats along with increases in the display of spatial memory and memory retention (35). According to Taihuttu *et al.* (2016), the effect of ethanol extracts of Gotu kola with various doses (150 mg/kg and 300 mg/kg) on the cell proliferation in the medial prefrontal cortex of adult rats after administration of electrical stress have not been found (36).

In this study, we quantified not only BDNF protein level and mRNA expression but also the TrkB mRNA expression in the hippocampus. The bond between BDNF and TrkB receptors leads to the dimerization and auto-phosphorylation of tyrosine residues on the intracellular receptor domain and activation of cytoplasmic signalling pathways (37). The BDNF-TrkB complex triggers the recruitment of adapter proteins and enzymes involved in BDNF signal transduction. Mitogen-associated protein kinase (MAPK), phospholipase C-γ (PLC-γ), and phosphatidylinositol-3-kinase (PI3-K) are the three main signaling molecules that mediate neurotrophin signals (5, 37).

The results of the Western blot analysis indicated the involvement of ERK1/2, but not p38 and JNK as the main pathway which mediates the effects of BDNF/TrkB in our study. Immunoblotting quantification revealed up-regulation of ERK1/2 protein expression in the CeA 600 group compared to the control group (Figure 2). This finding demonstrated that in the group which was given a 600 mg dose of *C. asiatica*/kg body weight followed by electrical stress, increased ERK1/2 occurred as down-stream of BDNF-TrkB signalling. 

The ERK1/2 had been reported to play role as down-stream signalling of the BDNF-TrkB complex which correlates with long-term potentiation (LTP), and the learning and memory processes. Moreover, the induction of LTP through titanic stimulation (1-100 Hz) caused the activation of the ERK signals and the administration of ERK inhibitors inhibits LTP in the rats. Inhibition of MAPK/ERK phosphorylation was shown to block phosphorylation of both CREB and Elk-1, and resulting in a rapidly decaying LTP (38). Induction of MAPK/ERK kinase inhibitor (U0126) intraventricular caused a significant increase in the amount of time needed by the rats to identify new and old objects (39). A study by Davis *et al.* (2003) showed that LTP which occurs in the dentate gyrus of the hippocampus is due to phosphorylation and Elk 1 transcription factor activation and could be inhibited by the MEK inhibitor (40). One of the transcription factors that play a role in learning and memory processes is cAMP response element binding (CREB) which has a crucial roles in learning and memory, especially through participation in adult hippocampal neurogenesis (41, 42). 

ERK is known to play role in other brain areas, such as inhibition of MAPK/ERK and inhibited LTP induction in anterior cingulate cortex of mice. However, the ability to sustain LTP was undisturbed (43). MAPK/ERK activation is required for the memory consolidation process through Pavlovian fear conditioning and synapse plasticity in the amygdala (44). ERK, but not JNK also is involved in BDNF inducing long-term potentiation in intact adult hippocampus, with CREB coupling (45). Siganlling pathways of the BDNF/ERK corelates to the cell differentiation and survival of the immature progenitor neuron leading to hippocampal spontaneous recovery after trimethiltin (TMT)-induced neurodegeneration (46). BDNF and ERK activation was also shown after Oleoylethanolamide (OEA) treatment in excessive consumption of alcohol and cannabis during adolescence (47). This study supports activation and contribution of ERK in ameliorating of BDNF-TrkB pathway in chronic electric stress with CeA treatment. 

## Conclusion

The hippocampal BDNF signalling through TrkB receptor and MAPK (ERK1/2) pathway have significant contribution in the amelioration of memory performance after *C. asiatica* treatment in chronic electrical stress model in rats. 
